# Extracellular Vesicles: A Novel Opportunity for Precision Medicine in Respiratory Diseases

**DOI:** 10.3389/fmed.2021.661679

**Published:** 2021-07-23

**Authors:** Jonathan M. Carnino, Zhi Hao Kwok, Yang Jin

**Affiliations:** Division of Pulmonary and Critical Care Medicine, Department of Medicine, Boston University Medical Campus, Boston, MA, United States

**Keywords:** extracellular vesicles, precision medicine, respiratory diseases, exosome, lung disease

## Abstract

Extracellular vesicles are membrane-bound nanoparticles secreted by cells which play a well-known role in cell to cell communication. The most update to date nomenclature categorizes extracellular vesicles based on their relative size, protein markers, and/or the cell type of origin. Extracellular vesicles can be isolated from biological fluids using a variety of methods, including but not limited to, ultrafiltration, size-exclusion chromatography, differential ultracentrifugation, density gradient centrifugation, precipitation-based methods, and immunoaffinity capture. These nanovesicles carry distinct “cargo,” made up of biomolecules such as nucleic acids, lipids, and protein, which is delivered to nearby target cells. The “cargo” profile carried by extracellular vesicles is critical in their role of communication and resembles the physiological status of the cell they originated from. For the purpose of this review, we will focus on the miRNA cargo. Extracellular vesicle-miRNA profiles hold the potential to be used in diagnostic panels for a variety of diseases through a novel method known as “liquid biopsy.” In addition to this, extracellular vesicles may serve as a potential method to deliver drugs to specific cells within the body. This mini-review provides background into what extracellular vesicles are, methods of isolating these nanoparticles, their potential use as a biomarker and drug delivery system for precision medicine, and a summary of the current literature covering the role of some extracellular vesicle-cargo's in various pulmonary diseases.

## Introduction

### Biology of Extracellular Vesicles

**Figure 1:**
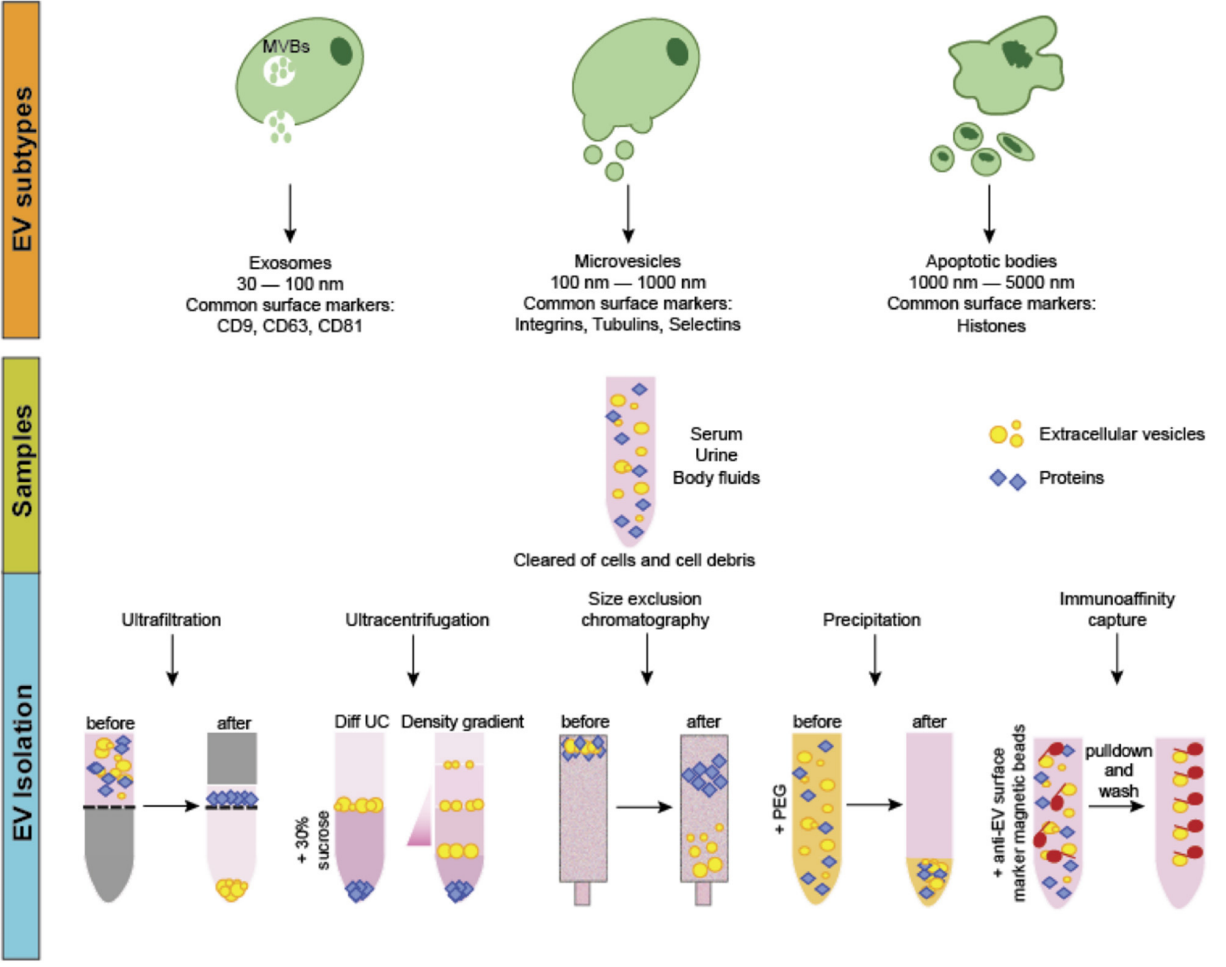
EV classification, biogenesis and isolation. EVs can be classified into three main categories. Apoptotic bodies (ABs) are the largest in size and are released from cells undergoing apoptotic cell death. Microvesicles (MVs) are considered as medium-sized EVs generated *via* membrane budding and shedding. Exosomes are the smallest EVs formed as multivesicular bodies. Production and secretion of microvesicles and exosomes generally involves specialized molecular machineries such as the endosomal sorting complexes required for transport and lipid rafts. EVs can be isolated *via* several methods including ultrafiltration, size-exclusion chromatography, differential and density gradient ultracentrifugation, precipitation and immunoaffinity capture. Often, samples are first centrifuged to remove cells and cellular debris. For ultrafiltration and size-exclusion chromatography, EVs are isolated through nanomembrane filters based on their sizes. In ultracentrifugation and density gradient centrifugation, different EV types are sequentially isolated based on physical properties such as density and sedimentation rate. For precipitation-based methods, the addition of water-insoluble polymers force and concentrate the EVs from the other components in the samples. Immunoaffinity capture method involves isolating particular EV types based on the use of magnetic beads coupled with antibodies that bind specifically to a known EV-specific surface marker for separation.

Extracellular vesicles (EVs) are cell-derived, membrane-bound nanoparticles which are secreted by nearly all cell types and play a known role in cell to cell crosstalk (1–3). EVs contain and transport a variety of “cargo” including lipids, proteins, and nucleic acids (1). Based on their physical sizes, biogenesis, and surface markers, EVs were previously classified into three main categories, namely the apoptotic bodies (ABs), microvesicles (MVs) and exosomes (Figure 1). ABs are regarded as secreted vesicles by cells undergoing apoptosis and typically range 1,000–5,000 nm in size (4). Microvesicles, on the other hand, are generated by the outward budding and consequent pinching of the plasma membrane and measure 100–1,000 nm in size (5). Lastly, exosomes are formed from the maturation of intraluminal vesicles as multivesicular bodies before their fusion with the plasma membrane for secretion. The size of exosomes typically ranges between 30 and 100 nm (Figure 1) (3, 5, 6). Recently, the lack of consensus on specific surface markers for these three categories, coupled with the overlap in their physical sizes, prompted the discontinuation of the aforementioned nomenclature for EV classification. Instead, guidelines set by the International Society of Extracellular Vesicles suggest that EVs can be termed based on: (a) physical characteristics of EVs, such as size [“small EVs” and “medium/large EVs,” with ranges defined, for instance, respectively, <100 nm or <200 nm (small), or >200 nm (large and/or medium)] or density (low, middle, high, with each range defined); (b) biochemical composition (CD63+/CD81+-EVs, Annexin A5-stained EVs, etc.); or (c) descriptions of conditions or cell of origin (podocyte EVs, hypoxic EVs, large oncosomes, apoptotic bodies)” (7, 8).

Over the years, several mechanisms have been proposed and elucidated for EV generation. Large EVs such as the ABs are usually produced *via* the disintegration of the plasma membrane during the process of apoptosis and may contain the fragmented organelles and nucleus. The production of small EVs (mainly MVs and exosomes) however, often requires coordinated molecular processes involving various protein machineries. For example, lipid raft proteins were demonstrated to mediate MV generation in the direct budding and pinching of the plasma membrane while the endosomal sorting complexes required for transport machinery was shown to participate in the multivesicular body-mediated generation of exosomes (5, 9). Despite the distinction in these mechanisms, generation of small EVs may employ multiple mechanisms involving overlapping molecular machineries that can be influenced by factors such as cell physiology, cell type, presence of exogenous stimuli and cargo content. Particularly, the differential cargo contents, as well as the modifications of these cargo contents including the post-translational modifications of EV cargo and surface proteins (10), observed across EV populations may provide an opportunity for the tracing of their origins (or their “mother” cells). Certainly, the identification of these sources of diseasing-causing EVs, alongside the ability to characterize distinct EV cargo signatures, may aid in the development of novel diagnostic and therapeutic strategies for some of the EV-associated diseases, especially in the era of precision medicine.

As discussed earlier, EVs are generated *via* multiple mechanisms which were previously used for EV classification (ABs, MVs, and exosomes). Despite the differences between each pathway, one critical characteristic of all EVs released is that their lipid-bound membrane resembles that of their host cell (1, 9). This trait is critically important in the ability to use “liquid biopsy” as a diagnostic method. The term “liquid biopsy” was originally developed and earned interest within the field of oncology (11). This method served as a diagnostic tool to detect, analyze, and monitor cancer by analysis of biological fluids such as urine and blood (11). However, in more recent times, this term has taken on a broader meaning representing a potential diagnostic tool for many more diseases. With the advancement of EV-cargo analysis and continuous efforts to discover EV-cargo represented in different diseases, liquid biopsy through EVs holds great potential for the future. For example, EVs can be easily collected and isolated from most biological fluids; for example, urine, blood, bronchoalveolar lavage fluid (BALF), nasal lavage, pleural fluid, etc. (1). Most studies currently analyze the EV-cargo, most commonly the miRNAs present and their relative expression to control patients. By further studying these isolated EVs and using nanoFACS to examine the cell membrane markers present on their exterior, we can understand the cell type they originated from. For example, a patient may come into the clinic with various pulmonary symptoms consistent with a variety of diseases. Blood could be collected and total EVs isolated from the sample. We may find that a population of exosomes present in the blood contain miRNA expression profiles consistent with acute inflammation and necrosis. Further analysis of these EVs may show the protein markers expressed on these exosomes come from type II lung epithelial cells, thus narrowing down the list of possible illnesses and directing the clinician to a possible diagnosis. This method of diagnosis, which may be termed “liquid biopsy,” would likely be much quicker than the typical tests performed to diagnosis such an illness. In summary, EVs hold the potential to be used as a diagnostic biomarker given their ability to be analyzed for certain cargo-profiles consistent with different diseases; and also, the capability to analyze their surface protein markers to determine the organ/tissue/cell type which they originated from.

Additionally, EVs have the potential to be used to deliver a drug to specific cells in the body. Free EVs can be taken up by nearby cells through a variety of pathways including clathrin-dependent endocytosis, caveolin-mediated uptake, phagocytosis, micropinocytosis, and lipid raft-mediated internalization (12). Both *in vitro* and *in vivo*, methods have been developed to confirm the delivery and uptake of EVs using fluorescently tagged surface proteins (13, 14). These advancements have a novel use for precision medicine as there is the potential to introduce drug-containing EVs to a patient's bloodstream, or by another method, that selectively deliver their contents to a specific tissue or cell type in the body. This specificity would greatly reduce the risk of toxicity and allow for higher doses of therapeutic agents to be given, significantly.

## Analytic Procedures to Study Extracellular Vesicles

At present, the common methods to isolate EVs include size-based separation (such as ultrafiltration and size-exclusion chromatography), differential ultracentrifugation and density gradient ultracentrifugation, precipitation-based and immunoaffinity capture methods (Figure 1) (15, 16). These methods offer both advantages and disadvantages, depending on factors like sample amount, sample source, desired EV yield and purity, as well as the downstream analysis procedures.

### Ultrafiltration and Size-Exclusion Chromatography

Ultrafiltration involves the usage of nanopore membrane filters made of polyethersulfone or polyvinylidene difluoride that allow an approximate 50–100 kDa molecular mass cutoff (17). For SEC, passing the samples through sepharose-packed columns allows for the separation of the EVs based on the vesicular size differences. Both methods can be particularly useful for the of highly purified small EV fraction with great reproducibility and for diluted samples like urine and cell culture media (18). However, the processing time for these methods is relatively long, making their scalability for routine clinical applications limited.

### Differential Ultracentrifugation and Density Gradient Centrifugation

These approaches are based on the differential density and sedimentation rates of EVs from other components in the biofluids. For differential ultracentrifugation, a series of low-speed centrifugations (3,000–20,000 × g) are initially performed to remove large particles like cell debris and platelets as well as to isolate the ABs and MVs. Following which, ultracentrifugation at ~100,000 × g typically yields the smaller EVs such as the exosomes. Density gradient centrifugation relies on using columns containing solutions of different densities to sequentially isolate the different EV fractions with differential densities at specific column layers. This method is typically performed following ultracentrifugation for a purer separation of the small EV fractions (19). While allowing greater purity of the isolated fractions, the combination of these two methods often requires higher starting sample volumes and reduces the yield of intact particles probably as a result of the increased risk of EV rupturing owing to the high centrifugal speeds (20). Furthermore, they are time-consuming while requiring specialized, costly equipment and hence, not suitable for processing large number of samples for clinical applications.

### Precipitation-Based Methods

EV separation *via* precipitation involves the use of water-insoluble polymer mixtures containing chemicals like polyethylene glycol to force the insoluble components out from the samples (21, 22). Currently, several precipitation-based EV separation kits are commercially available. As this method is relatively quick, convenient, and requires no specialized equipment, it is potentially feasible to be scalable for processing large number of samples in routine clinical applications. Furthermore, recent studies have demonstrated an increased yield and enhanced detection of the alterations of markers in prostate cancer-derived small EVs (23). The disadvantage of this method, however, is the reduced purity of the specific EV types isolated since both large and small EVs, as well as abundant proteins in the samples can often be co-precipitated.

### Immunoaffinity Capture

Lastly, this method relies on the presence of known, specific surface protein markers of the different EV types. Antibodies against these markers are conjugated to magnetic beads to allow the specific binding and subsequent separation of these EVs by magnetic techniques. This method was shown to be more efficient and economical as compared to precipitation-based EV separation (24). It is useful for isolating EV subpopulations such as tumor-specific small EVs when the specific markers have been identified. However, owing to the sophisticated processing steps involved and relatively low yield, this method is limited for its clinical applications since it requires huge amount of starting material, as well as skilled operators with strong technical expertise.

## EVs as a Potential Biomarker and Therapeutic Target to Enhance Precision Medicine

### EV-miRNAs as a Biomarker

EV-miRNAs have been of interest lately as a possible diagnostic biomarker for diseases given their ability to reflect host cell status and condition (25, 26). Furthermore, EV-miRNAs are protected by the EV lipid bilayer, extending their half-life and making them more stable diagnostic markers for separation than free-miRNAs in the body (25, 27). As mentioned earlier, EVs display the same membrane markers as their host cell. This means that through EV characterization and miRNA, protein, or lipid profiling, it is possible to detect a cell showing pathological status and know the cell type or tissue under stress. Furthermore, EV-cargo may also be cell specific; one example, OMVs (bacteria-derived MVs) carry bacterial specific protein, thus, if detected in bodily fluids, will indicate the source of the infection. Another example, OMVs may carry antibiotic-resistant proteins, which if detected, may influence clinical decisions to change an antibiotic dose/choice. Lastly, modification to EV cargo (proteins/nucleotides) appears to be dependent to specific stimuli or disease processes. For example, oxidative stress was reported to induce caveolin-1 tyrosine 14-phosphorylation and this is detectable in MVs (28). For these reasons, EVs have great potential to be specific and accurate diagnostic biomarkers.

### EV-miRNAs as a Therapeutic Agent

EVs are recognized as a novel way to transport drugs or therapeutic agents (29, 30). Given that EVs are made up of a protecting and stabilizing lipid-bilayer, they can successfully transport cargo to recipient cells, giving them great promise as a next generation method of drug delivery (31). In addition, their similar composition to a normal cell membrane and the surface proteins they possess make them easily absorbed by target cells (30, 32). MiRNA- and mRNA-based drugs have been difficult to develop historically due to stability issues. However, with an EV-based delivery system protecting the therapeutic agents within their wrapped vesicles, drug-stability issues are solved (31). Moreover, since EVs are derived from a host they presumably will not be induce immune responses when delivered. In addition, although still under investigation, EV-surface proteins may have the ability to even guide drug delivery to specific organs/tissues (31). Furthermore, the ability of EVs to bypass the blood-brain barrier adds to its promise as a useful drug delivery system that should be further established (33). Lastly, EVs are a versatile method of drug delivery because they can be delivered from many different routes: intravenous, intraperitoneal, inhalation, intramuscular, etc. Particularly for delivery by inhalation, we can potentially use EVs to target the large airways *via* MVs and alveolar sacs *via* exosomes; as different sized EVs will precipitate into different regions of the airway.

In recent years, research has begun to focus on the role of EVs in many diseases and their potential role as a diagnostic biomarker and/or therapeutic agent. Below we offer a summary on the latest studies in on the role of EVs in respiratory diseases.

## EVs in Respiratory Diseases: Current Diagnostic and Therapeutic Applications of EVs

### EVs in Airway Diseases

Asthma is a chronic respiratory illness characterized by airway narrowing in response to a variety of stimuli (34). One study which analyzed the cargo miRNA profile of exosomes in patients with asthma found significant alterations in BALF exosomal miRNA for 24 miRNAs, including the let-7 and miR-200 families (34). Notably, BALF collection from patients is an overly invasive method to diagnose asthma, however this study proved EV-miRNA profiling can be used diagnostically in the disease. Another report analyzed exosomes in nasal lavage from patients with asthma and chronic rhinosinusitis (35). They found nasal lavage exosomes to induce the migration of innate immune cells, and that these exosomes carried a reduced number of barrier and antimicrobial proteins compared to healthy patients (35). Another report, which was notably the first group to detect miRNAs from exhaled breath condensate, hypothesized that the ability to detect them can be contributed to the stability offered from their encapsulation within exosomes (36). Testing in patients with asthma and tuberculosis, compared to healthy individuals, they found that miRNA profiles isolated from exhaled breath condensate reflected general inflammatory processes and/or epithelial damage (36). These findings show EVs collected from a much less invasive procedure such as nasal lavage, or even from exhaled breath, can be analyzed and have clinical implications for disease progressions.

Chronic obstructive pulmonary disease (COPD) is the cumulative name for a number of diseases that share the common element of limited expiratory airflow (37). The American Thoracic Society defines COPD as chronic bronchitis and emphysema (38). The role of EVs in COPD has been of interest lately and many reports have focused on understanding the significance of EVs in the disease processes. Numerous EV-miRNAs have been reported to be upregulated in the BALF, serum, and plasma of COPD patients (39–41). Specifically, analysis of exosomal-miRNA from BALF shows an upregulation of miR-223-3p,−223-5p,−338-3p,−1469,−204-5p, and−618 (41). Analysis of exosomal-miRNA from serum shows an increased expression of miR-21 and MV-miRNA from plasma shows an increased expression of miR-191,−126, and−125a (39, 40). These reports build on the case that EV-miRNA from biological fluids can serve as a useful tool in diagnosing patients with COPD.

Idiopathic pulmonary fibrosis (IPF) is a chronic and progressive type of interstitial lung disease characterized by fibrosis and deteriorating lung function (42). Compared to asthma and COPD, there has been much less research into EV-miRNA diagnostic markers for IPF. One recent report found serum EV-miR-21-5p to be elevated in patients with IPF compared to control (43). Although these findings suggest serum-EV-miR-21-5p may be a suitable diagnostic marker for IPF, further studies are necessary to uncover additional EV-miRNAs expressions altered in the setting of IPF.

### EVs in Lung Parenchyma Diseases

Acute lung injury (ALI) is a devastating illness characterized by non-cardiogenic pulmonary edema, vascular leakage, inflammation, and lung epithelial injury (13). Extensive research been done to reveal miRNAs involved in ALI and inflammation, however very few groups have studied EV-miRNAs in these settings. Recently, increased expression of BALF MV-miR-185-5p was reported to induce necroptosis and apoptosis in type II lung epithelial cells (13). Another recent study suggests increased expression of MV-miR-223/142 plays a role in the pathogenesis of lung macrophage-mediated lung responses and this upregulation could be detected in both BALF and serum (44). Furthermore, MV-miR-17/221 and MV-miR-320a has been shown to induce macrophage recruitment and subsequently contribute to lung inflammation (45, 46). Additionally, increased expression of the EV-miR-466 family has been shown to exacerbate inflammation *via* the NLRP3 inflammasome pathway (47). Of note, all of these mentioned reports were in mouse models and there is a significant lack of clinical research into EV-miRNAs in ALI and inflammation. Due to the limited studies into EV-miRNAs involved in ALI and inflammation, further work is necessary to completely uncover the role EV-miRNAs play in this disease and develop a diagnostic panel that could be used clinically.

### EVs in Lung Cancer

Lung cancer is the primary cause of cancer-related deaths in the world and accounts for about 25% of all cancer mortality (48). In one clinical study including 21 patients with lung cancer and 25 control patients, EV-miRNAs isolated from pleural lavage showed an upregulation of EV-miR-150-5p,−27a-5p,−21-3p,−1249-3p, and−485-5p and a downregulation of EV-miR-144-5p,−1-3p,−584-5p,−133b,−451a,−199a-5p,−20b-5p,−181c-5p, and−30e-5p in cancer patients (49). This study showed that in the clinical setting, EV-miRNAs isolated from pleural fluids are a potential source of biomarkers for lung cancer. These results have promise for being used to develop a potential diagnostic panel for lung cancer. An additional study which analyzed EV-miRNAs from plasma in patients with lung cancer, found an increased expression of EV-miR-21,−191, and−192 in patients with lung cancer (50). A useful and interesting addition to these studies would've been to see how the level of expression correlated with prognosis, which would allow an EV-miRNA panel to be used by physicians in providing patients with an accurate prognosis.

### EVs in Pleural Diseases

Malignant Pleural Mesothelioma (MPM) is a form of cancer manifesting in the pleural cavity, and its incidence is strongly correlated with a past asbestos exposure (51). The relationship between asbestos exposure and MPM is well-documented and has a latency time between 30 and 40+ years (52). One recent clinical study, which included 23 MPM patients and 19 cancer-free subjects with a history of asbestos exposure, suggested that EV-miR-103a-3p and miR-30e-3p isolated from plasma was upregulated in MPM patients compared to the control subjects (53). This study has been the only one carried out to analyze EV-miRNA profiles in patients with MPM. Additional pleural diseases such as pleurisy, pleural effusion, pneumothorax, and hemothorax lack any research into EV-miRNAs related to these illnesses (Table 1).

**Table 1:** Summary of reported EV-miRNAs in respiratory diseases.

**Disease**	**EV-miRNAs involved**	**References**
Asthma	Altered expression of BALF exosomal-miRNAs including let-7 and miR-200 families. Nasal lavage exosomal-miRNAs induce immune cell migration. Exhaled breath condensates contain pro-inflammatory EV-miRNA repertoires.	(34–36)
COPD	Exosomal-miR-223-3p,−223-5p,−338-3p,−1469,−204-5p, and−618 upregulated in BALF. Plasma exosomal-miR-21 upregulated and MV-miR-191,−126, and 125a upregulated.	(39–41)
IPF	Serum EV-miR-21-5p elevated.	(43)
ALI	Increased BALF MV-miR-185-5p induces necroptosis and apoptosis. Lung macrophage-mediated lung responses modulated by BALF and serum MV-miR-223/142. MV-miR-17/221 and MV-miR-320a induce macrophage recruitment and lung inflammation. EV-miR-466 worsens lung inflammation *via* the NLFP3 inflammasome pathway.	(13, 44–47)
Lung cancer	Pleural lavage from patients has shown an upregulation of EV-miR-150-5p,−27a-5p,−21-3p,−1249-3p, and−485-5p and a downregulation of EV-miR-144-5p,−1-3p,−584-5p,−133b,−451a,−199a-5p,−20b-5p,−181c-5p, and−30e-5p. Additionally, the plasma of lung cancer patients have an increased expression of EV-miR-21,−191, and−192.	(49, 50)
MPM	Upregulation of plasma EV-miR-103-3p and miR-30e-3p.	(53)

## Future Perspectives

The intrinsic properties of EVs, i.e., the small sizes that allows their passive entry into various organs and tissues, the encapsulated structure that provides a shield for their cargo load against degradation and the modifications of the EV surface proteins that enable enhanced specificity for delivery to certain tissues, make them a highly valuable drug delivery option as novel therapeutic strategies for diseases. With a diverse cargo load of nucleic acids, proteins and lipids, a comprehensive profiling of these molecular signatures can represent “liquid biopsies” of individuals with certain pathological diseases and further developed into unique biomarker library panels in the context of precision medicine. Advances and standardization in EV separation and characterization methods, coupled with increasing knowledge of EV biogenesis and uptake mechanisms, will no doubt propel our leverage of EVs as invaluable diagnostic and therapeutic tools in the era of personalized medicine.

## Author Contributions

JC drafted the manuscript. ZH drafted the figure and legend. YJ designed the outline of the topic and helped on revising the manuscript. All authors contributed to the article and approved the submitted version.

## Conflict of Interest

The authors declare that the research was conducted in the absence of any commercial or financial relationships that could be construed as a potential conflict of interest.

## Publisher's Note

All claims expressed in this article are solely those of the authors and do not necessarily represent those of their affiliated organizations, or those of the publisher, the editors and the reviewers. Any product that may be evaluated in this article, or claim that may be made by its manufacturer, is not guaranteed or endorsed by the publisher.
